# Performance Evaluation of Quantum-Based Machine Learning Algorithms for Cardiac Arrhythmia Classification

**DOI:** 10.3390/diagnostics13061099

**Published:** 2023-03-14

**Authors:** Zeynep Ozpolat, Murat Karabatak

**Affiliations:** Department of Software Engineering, Firat University, 23119 Elazig, Turkey

**Keywords:** electrocardiography classification, quantum computing, machine learning, quantum support vector machine

## Abstract

The electrocardiogram (ECG) is the most common technique used to diagnose heart diseases. The electrical signals produced by the heart are recorded by chest electrodes and by the extremity electrodes placed on the limbs. Many diseases, such as arrhythmia, cardiomyopathy, coronary heart disease, and heart failure, can be diagnosed by examining ECG signals. The interpretation of these signals by experts may take a long time, and there may be differences between expert interpretations. Since technological developments are intertwined with the medical sciences, computer-assisted diagnostic methods have recently come forward. In computer science, machine learning techniques are often preferred for automatic detection. Quantum-based structures have emerged to increase the machine learning algorithm’s speed and classification performance. In this study, a quantum-based machine learning algorithm is applied to classify heart rhythms. The ECG properties were converted to qubit structure using principal component analysis (PCA). The resulting qubits are classified using the quantum support vector machine (QSVM) algorithm. Quantum computer simulation over Qiskit was used for classification studies. Within the scope of experimental studies, comparisons between classical SVM and QSVM were made using different data amounts and qubit numbers. In the results of the analysis, classical SVM achieved 86.96% accuracy, and QSVM achieved 84.64% accuracy. Despite the fact that the entire dataset was not used due to various limitations, these successful performances were achieved. Classification of medical data such as that from ECG has shown that quantum-based machine learning frameworks perform well despite current resource constraints. In this respect, the study includes essential contributions to the use of quantum-based machine learning methods on signal data in medicine.

## 1. Introduction

Early diagnosis of cardiovascular diseases is critical in determining treatment and preventing important risks, such as mortality. Rhythm disorders (arrhythmias) occur in the heart due to cardiovascular diseases. Electrocardiography (ECG) is the general diagnostic method used to diagnose arrhythmias. Electrical signals of the heart are recorded with an ECG and interpreted by experts through observation. One of the main reasons that many diseases cannot be treated is the lack of timely intervention. Due to factors such as a rise in patient numbers, the inadequate quality of medical equipment, and a shortage of doctors, early diagnosis may be delayed. In addition to these parameters, the results of the analyses take a long time [1]. Since the heart is one of the vital organs in the body, there is a great deal of research in computer science regarding heart disease. Li et al. [2] have developed a custom networking structure called a Beat-aligned Transformer (BaT) to take advantage of the repetitive features of ECG data. The concept of “deep learning” [3,4], in which both classification and feature inference have coexisted recently as a result of improvements in machine learning, has rapidly become widespread. In ECG analysis, deep learning architectures have also provided good performances. Baloglu et al. [5] applied a convolutional neural network (CNN) for the diagnosis of myocardial infarction (MI) by processing the 12-leads ECG signal. As a result of their analysis, high performance results were achieved. In another study for MI, Yıldırım et al. [6] developed a deep neural network (DNN) trained with surface ECG to detect clinical MI disease in people. The superiority of the developed model was demonstrated by comparing it with Q-wave analysis. Similarly, it has produced positive results in the treatment of atrial fibrillation (AF) caused by cardiovascular conditions [7,8].

Computational power in the advancement of machine learning methods is mostly based on hardware, and parallel software improvements produce significant impacts. The purpose of machine learning is to increase speed and performance. An outcome can be obtained more quickly if all potential solutions are calculated. One of the main reasons for entering the quantum world is that a resolution can be made quickly by following a different path for each possibility. Quantum physics, one of the important subjects of physics, is a field that contains theories about the entire subatomic microscopic particle system. It has been researched from a very different perspective in recent years as a result of its contributions to the world of informatics [9]. Unlike the bits used in classical computers, qubits are used in quantum computers. A qubit can take the values 1 or 0, or it can be both 1 and 0 simultaneously. As a result, quantum computers can compute multiple probabilities at the same time [10].

In recent years, quantum-based machine learning algorithms have rapidly become popular in the literature. Maheshwari et al. [11] evaluated analysis results by applying both classical and machine learning algorithms to diabetic patient data. Gupta et al. [12] compared deep learning (DL) and quantum machine learning (QML) algorithms in another study on diabetes. Zhang and Ni [13] have suggested in their research that some of the supervised and unsupervised machine learning algorithms based on the quantum circuit model focus on the quantum base. As a result of their studies, they determined that quantum algorithms show a speed-up in results compared to their classical versions. Blance and Spannowsky [14] aimed to increase performance in solving classification problems by combining quantum computing methods with classical neural network techniques.

In this study, the use of quantum-based machine learning algorithms in ECG analysis, which is one of the important problems in the medical field, is proposed. For this purpose, an ECG dataset [15] containing four different rhythms was analyzed using both the classical and quantum-based support vector machine (SVM) methods. For quantum SVM, qubits were created with principal component analysis (PCA), a size reduction algorithm, in parallel with the existing hardware resources. Comparisons between the performances of classical SVM and quantum support vector machine (QSVM) were examined, with both the number of data points and qubit numbers increasing at different rates. 

The organizational structure of this study is as follows. In Section 2, the materials and methods are introduced. In Section 3, details about the experimental studies are given. Section 4 and Section 5 conclude our study; this includes the discussion and results, respectively.

## 2. Materials and Methods

**Figure 1:**
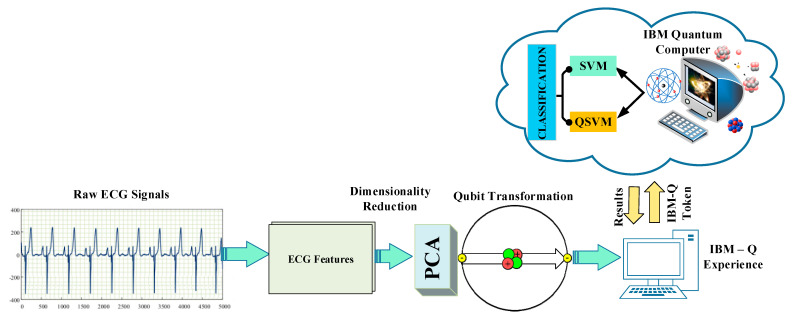
Illustration of the proposed methods.

In this article, analyses were performed with a quantum computer simulator using a dataset of ECG signals. The dataset was converted from bit form to qubit form [16]. Following the data preparation for analysis, a classification procedure was carried out using the QSVM method from open-source Qiskit codes [17]. The classical SVM algorithm was applied to the dataset in qubit form, which was labeled by reducing its size. The purpose of this was to compare the performances of the QSVM and SVM algorithms on the same data. A block representation of the materials and methods used in the study is given in Figure 1.

### 2.1. Arrhythmia Dataset

In this study, the dataset created by Zheng et al., which contains ECG data from 10,588 patients, was used [15]. This dataset was created from the Chapman University and Shaoxing and Ningbo People’s Hospital (Chapman) database. The dataset includes raw ECG signals from 12 leads and 11 clinically obtained ECG features. These features are: ventricular rate (VR), atrial rate (AR), QRS duration (QRSD), Q interval, QT corrected, R axis, T axis, QRS count, Q onset, Q offset, and T offset.

The noise-free ECG dataset consists of 12-lead ECG signals sampled at 500 Hz and categorized by 11 rhythm classes. These are atrial flutter (AF), atrial fibrillation (AFIB), atrial tachycardia (AT), atrioventricular node reentrant tachycardia (AVNRT), atrioventricular reentrant tachycardia (AVRT), sinus irregularity (SI), sinus atrium to atrial wandering rhythm (SAAWR), sinus bradycardia (SB), sinus rhythm (SR), sinus tachycardia (SINT), and supraventricular tachycardia (SVT). Murat et al. [18], using this data in their study, created 4 different class labels by converting classes with a small number of patients into groups that are related to each other. Information about these four rhythm classes combined is given in Table 1.

### 2.2. Proposed Method

**Figure 2:**
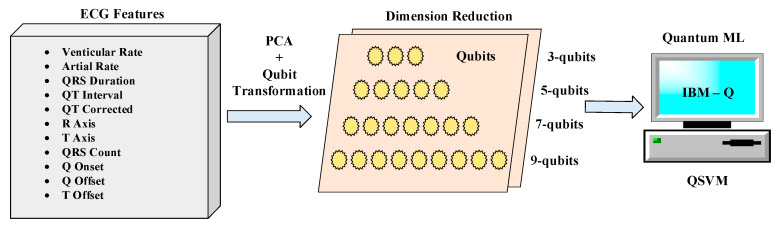
A block representation of our proposed method.

This study employs the recently popular quantum-based machine learning approaches in classifying heart arrhythmias. For this purpose, first, size reduction was performed on a determined dataset using the PCA technique. The features reduced by the dimension reduction technique were converted to qubit format and used in the classification stage of the QSVM algorithm. Apart from the QSVM algorithm, there are quantum classification algorithms such as quantum neural network (QNN) [19,20], quantum K-nearest neighbors (Q-KNN) [21,22], and quantum means (Q-Means) [23]. The QSVM algorithm is preferred in this study, because SVM is mainly used in classical algorithms in ECG classification. Since it is aimed at comparing classical and quantum-based ML, QSVM has been determined as the most suitable algorithm. A block representation of the proposed method within the scope of the study is given in Figure 2.

#### 2.2.1. Principal Component Analysis (PCA)

One of the earliest statistical tools, PCA is a method for converting oversized data into lower-dimensional data to reduce cost and speed. PCA maintains changes in data, allowing data to be expressed, at least at a loss, with fewer components than in its original state. In doing so, it aims to determine the best transformation and ensure that all the resulting components are independent of each other. In this direction, the variance of the data, eigenvalues, and eigenvectors are used while making calculations [24]. As a result of applied mathematical operations, it is ensured that the original dataset is expressed with different axes. Thus, more efficient analyses can be made, as the data is provided from a different perspective.

#### 2.2.2. Quantum Support Vector Machine (QSVM)

The orientation of computer science toward the quantum world has paved the way for the use of quantum-based programming in the classification stages. To run the SVM algorithm on a quantum computer, the algorithm must be rescheduled according to quantum rules [25]. The QSVM algorithm, a quantum adaptation of SVM, performs the computations for the basic SVM using the laws of quantum mechanics. Whereas classical SVM requires a graphics processing unit (GPU) or central process unit (CPU) to increase performance, QSVM uses the power of quantum software. When performing QML operations, classical data are converted into quantum data (qubits) to be used in quantum computers. Then, the processing steps required by the QML algorithm are applied. The result obtained is returned in the classical form [26].

#### 2.2.3. Experimental Setups

The ECG dataset used consists of 10,588 pieces of data. Since there is no access to quantum computers in the real environment, the dataset was run using the Qiskit framework in the existing computer architecture through the Anaconda package program. Since a real quantum computer cannot be used, the large amount of data creates a disadvantage in execution time. Data with a reduced original number of data are called a data case. The number of data is reduced using 7 different test sizes for data cases. The quantum computer system is still in development. Therefore, it can serve with limited qubits. PCA, one of the conversion methods, was used to avoid exceeding the qubit limit while preserving the structure of the features. SVM and QSVM performances were compared for 5 data cases: 3, 5, 7, 9, and 11. The attribute numbers given here as dim also indicate the qubit numbers simultaneously. 

**Figure 3:**
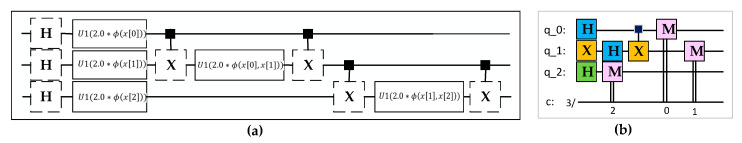
(**a**) ZZFeatureMap architecture. (**b**) A quantum circuit model.

The dataset, which initially had 11 dimensions, was reduced to 4 dimensions by applying PCA. Quantum simulation is provided in the classical computer with ZZFeatureMap [27], which is used for the qubits to enter the entanglement state. The circuit model of ZZFeatureMap is given in Figure 3a. A quantum circuit model for the case in which the number of qubits is determined to be 3 is shown in Figure 3b.

In this study, the Qiskit Library was used for the QSVM implementation [26]. Qiskit is an open-source, quantum-computing environment developed by IBM. Thanks to the Qiskit library, quantum experiments can be run on classical computers with quantum simulations. The Qiskit environment is used with the Python programming language. Various libraries must be added to Qiskit to perform quantum computations [28]. These libraries include Qiskit Terra, Qiskit Aer, Qiskit Ignis, Qiskit Nature, Qiskit Machine Learning, Qiskit Finance, and Qiskit Optimization. Many of these libraries were used during this study. Computer features used in the experimental studies are as follows: an Intel(R) Xeon(R) W-2245 with CPU@ 3.90GHz 3.91 processor, 32 GB RAM, and a NVIDIA Quadro RTX 4000 video card.

## 3. Experimental Results

This section presents the performance results of the QSVM method on heart rate data. QSVM and SVM algorithms are compared with two different scenarios, data states, and qubit numbers. In the first scenario planned, the performances of the QSVM algorithm were analyzed using different qubit numbers. Performance comparisons were made with the performances of the SVM algorithm for the same data cases. In the second scenario, the ways in which the change in the data space affects the performance of the QSVM algorithm are observed. The results obtained here are compared with the SVM algorithm as in the first scenario.

### 3.1. Scenario 1: Different Number of Qubits

**Figure 4:**
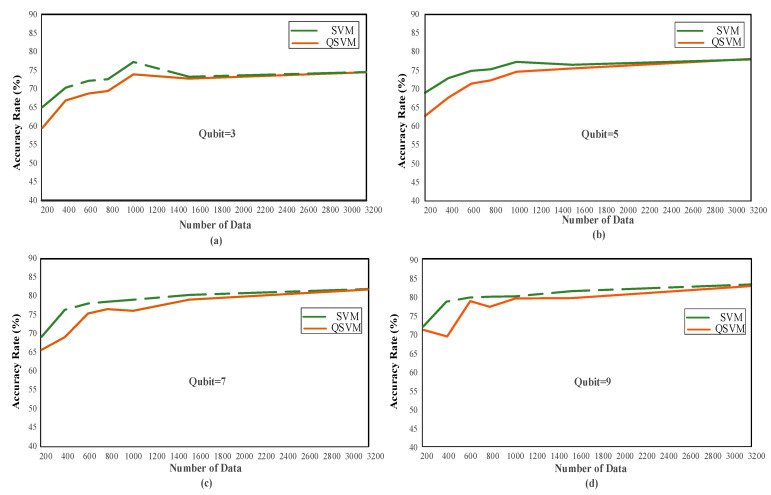
(**a**) The performance of data cases according to 3 qubits. (**b**) The performance of data cases according to 5 qubits. (**c**) The performance of data cases according to 7 qubits. (**d**) The performance of data cases according to 9 qubits.

PCA has been applied to 11 features (dimensions) of the ECG signals in the dataset used in this study and has been made available for the QSVM algorithm. The “feature_and_label_transform” plugin in Qiskit was used to re-label qubits after the PCA application. As a result of the applications, the dataset was reduced to dim 3, dim 5, dim 7, dim 9, and dim 11. The dimensions achieved after these reductions now constitute qubits. Figure 4 below gives the analysis visuals for the SVM and QSVM algorithms in different data states for four different qubit values: Q (3), Q (5), Q (7), and Q (9).

**Figure 5:**
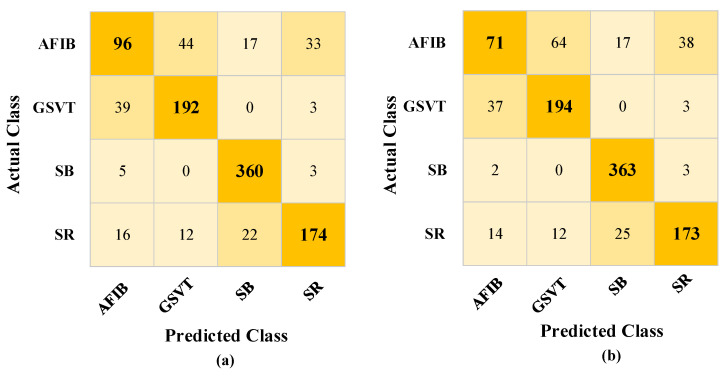
Confusion matrices obtained on test data for QSVM and SVM: (**a**) confusion matrix for QSVM, (**b**) confusion matrix for SVM.

When Table 2 is examined, it is determined that the QSVM performance is lower than or nearly equal to the general SVM. Among the results obtained for five qubits, it is seen that the QSVM algorithm is superior to SVM in the case of 3133 pieces of data. Even though there is only a very slight difference in this instance, it is projected that the QSVM algorithm will perform better under the right circumstances. Table 2 was obtained with average values based on 10 different cases. When the results in the Q (5) analyses for the 3133 pieces of data are examined, the QSVM achieved a performance of 80.90% accuracy, whereas the SVM showed a result of 78.84% accuracy under the same conditions. The confusion matrix of this situation is shown in Figure 5.

In Table 3, the precision, sensitivity, specificity, and F1 score performance metrics for the provided confusion matrices are given.

**Figure 6:**
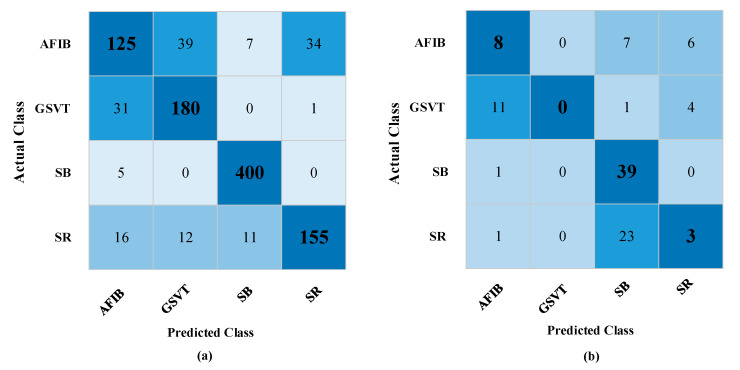
Confusion matrices obtained with quantum support vector machine (QSVM) classifier: (**a**) highest and (**b**) lowest accuracy.

When examining the tables and figures provided, performance increases as the amount of data increases. SVM achieved 83.17% accuracy, and QSVM achieved 82.73% accuracy as the highest performance (see Table 2) for Qubit = 9. The confusion matrix obtained from the QSVM algorithm is given in Figure 6a.

The lowest performance is observed when the number of data cases and qubits is the least. In the case of data number 209 for Qubit = 3, the worst results were obtained, with 65.09% accuracy with the SVM and 59.51% accuracy with the QSVM (See Table 2). The confusion matrix of these values is given in Figure 6b. The information, including the confusion matrices’ performance metrics, is shown in Table 4.

When analyzing the results, the reduction in qubits significantly affects the performance rate. As the number of qubits and size increase, the data attributes become clearer. This has increased the performance. At the same time, the increase in the sample used for the analysis also positively affects the performance.

### 3.2. Scenario 2: Different Amount of Data

**Figure 7:**
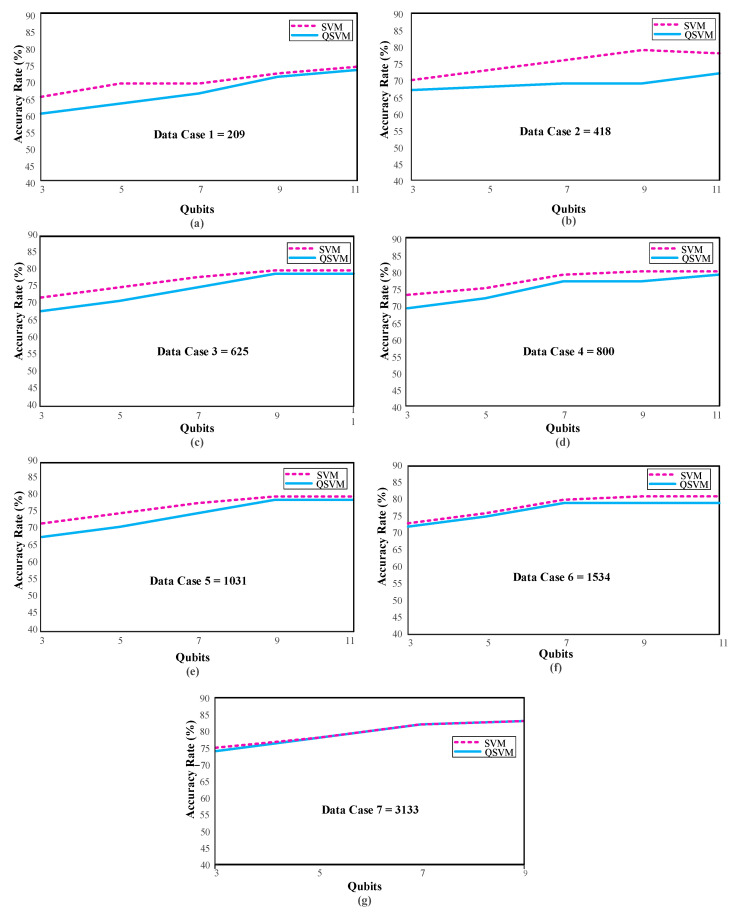
QSVM and SVM performances with different qubit numbers according to Data Case 1 (**a**), Data Case 2 (**b**), Data Case 3 (**c**), Data Case 4 (**d**), Data Case 6 (**e**), Data Case 6 (**f**), and Data Case 7 (**g**).

The arrhythmia dataset used in this study consists of 10588 pieces of patient data. The analysis takes a very long time due to the quantity of data. For this reason, data cases have been created using seven different test sizes while designing the data to obtain faster results by reducing the amount of data. Information about these data cases is given in Table 2. These results were obtained by calculating the mean and standard deviation of 10 different random state values. Figure 7 shows the performance variation in the qubits for each data case.

When Table 2 and Figure 7 above are examined, the highest performance for Data Case 7 was observed as 83.17 ± 1.38 with the SVM and 82.73 ± 1.13 with the QSVM in Q (9). Table 2 shows the performance of Q (9) in bold in all data cases. When the number of qubits is at its maximum and the number of data increases, the performance gradually increases, as the sample space to be used in the classification increases, as shown in Table 2. The main reason that the analysis could not be conducted with the total size of the dataset is that the studies take longer as the amount of data increases. For this reason, the amount of data was increased gradually. The experiment was carried out with a maximum of 3133 pieces of data. Further data analysis was not possible due to hardware restrictions.

## 4. Discussion

In this study, quantum-based machine learning algorithms are used to recognize heart rhythm classes automatically. Since quantum technology is a newly developing field, it has not been possible to develop a new algorithm due to structural deficiencies. The main purpose of the article is to observe the effects of parameters such as the qubit and data number on the performance using existing techniques. The results of various studies on similar datasets and the proposed method are compared in Table 5. Some of the studies in Table 5 are given performance comparison purposes, because they use the same dataset. In a study by Aziz et al. [29], in the SVM classification made for the SPNH database, the highest performance of 84.2% accuracy was obtained in the PR + RT + Age + Sex classes compared to other combinations. In MLP, on the other hand, 90.7% success was achieved under the same conditions. In an article by Sepahvand et al. [30], the model proposed by the authors is a CNN model, which is the teacher and student model. As a result of their studies, they obtained 98.96% accuracy in the teacher model and 98.13% accuracy in the student mode for seven rhythm classes. Faust et al. [31] achieved 99.98% success in the SPNH dataset with the ResNet deep learning algorithm used by the authors in their study. Dhananjay et al. [32] compared classical classification algorithms as well as their proposed model, the CatBoost model, in their study. Whereas 71% success was achieved with SVM, the success rate was 99% with the method suggested by the authors. Murat et al. [18] used deep learning algorithms to reduce property sizes using PCA with the SPNH dataset. They achieved a success rate of 84.06% in the SVM algorithm. Baygin et al. [33] presented a new classification model for the classification of ECG data, also affected by the homomorphically irreducible tree (HIT) problem with the SPNH dataset. The model they installed consisted of HIT model creation, maximum absolute pooling (MAP), Chi2 selective, and the SVM algorithm in classification. Their success rate was 97.18%. 

According to Table 5, it is observed that the QSVM algorithm performs poorly compared to other studies. The entire Chapman database could not be run in the QSVM algorithm, as the analysis took too long due to hardware deficiencies. Although only about 30% of the dataset was used in the study, the SVM algorithm achieved 86.96% success (for Data Case 2—Q (11)), and the QSVM algorithm achieved 84.64% success (for Data Case 7—Q (9)). Though the runtime takes hours for the case in which QSVM achieves 84.64% success, the SVM algorithm gives results in about 2 s under the same conditions (Data Case 7—Q (9)). These results show that the simulation environment creates a disadvantage in terms of time in quantum-based algorithms. However, it is clear that the QSVM algorithm competes with the classical SVM in terms of accuracy.

To the best of the authors’ knowledge, this study is the first to classify an ECG dataset using the QSVM algorithm. It aims to form a basis for future studies in the field of ECG. In addition to adding to the limited studies using QML in the literature, this study presents comparisons with classical SVM using the quantum-based SVM algorithm, which has not been used before in the classification of ECG data. The research shows that the QSVM method offers a comparable performance to the traditional SVM technique. It is predicted that the increase in the number of qubits, known as the size, and the increase in the amount of data in the classical environment will positively affect the algorithm. It is thought that this performance will become more competitive when the existing deficiencies are eliminated, and the entire dataset is run. For these reasons, the QSVM algorithm gives promising results in ECG diagnosis.

The main limitations of this study include the following. An IBM Q computer could not be used in this study due to the preprocessing processes applied. Instead, analyses were performed on a classical computer by creating a quantum environment. PCA was used because it has a qubit limitation. Due to the structural features of PCA, there may be a loss of features while reducing the size. This can negatively affect performance. The purpose of using quantum computers is basically to provide acceleration. However, the desired level could not be reached at the time of calculation, since the adaptation process with today’s computers has not yet been realized. Though the increase in the number of data causes an increase in performance, the results of the analyses take longer than with the classical algorithms. Since a quantum-based algorithm was used in this study, the entire dataset could not be used in the analysis due to hardware deficiencies. The primary purpose of this study was to demonstrate the usability of quantum machine learning algorithms that are under development for use with medical data such as ECG data. The analysis results given here were obtained using the Qiskit simulation environment. Analyses could not be performed in the real circuit due to issues with the use of the Noisy Intermediate-Scale Quantum (NISQ). Although a low performance was achieved according to state-of-the-arts studies, it is thought that this performance will improve when various limitations are overcome. If the same analyses were performed on the actual circuit, faster execution would probably be possible. The limitations will be eliminated in future studies, and analyses will be applied in the NISQ environment. Comparisons are limited due to the fact that we do not have the source codes and parameter values of the studies conducted on the same dataset [18,29,30,31,32,33]. For example, the effects of the amount of data on other methods have not been clearly demonstrated. 

In future studies, it is estimated that if the deficiencies of hardware are eliminated, and the entire dataset is used, both algorithms will provide better performances close to those of the classical studies existing. For performance comparisons, QML algorithms other than QSVM should also be used. It is known that the IBM Q computer is advantageous in terms of time in different datasets. After the Chapman dataset is made suitable, we aim to carry out analyses in an IBM Q real computer environment. After these studies, a detailed comparison with the results of the simulation environment will be presented.

## 5. Conclusions

In this study, quantum machine learning algorithms were used to classify arrhythmias. For a quantum-based machine learning algorithm to be applied, the dataset must first be converted to qubit format, known as quantum bits. A dimension reduction method, principal component analysis (PCA), has been applied. Reduced size classes were converted to qubit form with a converter. ECG features for 10,588 pieces of patient data were used in a quantum simulator on a classical computer. Different rates of dimensionality reduction were applied for the bits converted to qubit form. Classical SVM and QSVM algorithms were applied to this new qubit-format dataset, and the performances were compared. The QSVM algorithm’s performance seemed comparable to that of the traditional SVM. This performance of the QVSM on a limited number of ECG data is an important step in QML algorithms. Future research should aim to compare outcomes when various QML algorithms are applied to ECG signals.

## Figures and Tables

**Table 1:** Information on the merged ECG rhythm classes’ labels.

Merged Rhythms	New Class
AF + AFIB	AFIB
SVT + AT + SAAWR + SINT + AVNRT + AVRT	GSVT
SB	SB
SR + SI	SR

AF: atrial flutter, AFIB: atrial fibrillation, AT: atrial tachycardia, AVNRT: atrioventricular node reentrant tachycardia, AVRT: atrioventricular reentrant tachycardia, SI: sinus irregularity, SAAWR: sinus atrium to atrial wandering rhythm, SB: sinus bradycardia, SR: sinus rhythm (), SINT: sinus tachycardia, and SVT: supraventricular tachycardia.

**Table 2:** Accuracy rates with mean and standard deviations for QSVM and SVM classifier.

Method	Amount of Data						
	Data Case 1	Data Case 2	Data Case 3	Data Case 4	Data Case 5	Data Case 6	Data case 7
	209	418	625	800	1031	1534	3133
Qubit = 3							
SVM	65.09 ± 6.03	70.29 ± 2.95	72.14 ± 2.61	72.56 ± 2.60	77.23 ± 2.58	73.25 ± 2.17	74.50 ± 0.86
QSVM	59.51 ± 7.06	66.85 ± 5.08	68.73 ± 2.62	69.40 ± 2.56	73.88 ± 3.15	72.73 ± 2.26	74.46 ± 1.27
Qubit = 5							
SVM	69.03 ± 6.12	72.94 ± 3.73	74.90 ± 3.11	75.32 ± 2.44	77.29 ± 3.58	76.51 ± 2.05	77.94 ± 1.21
QSVM	62.78 ± 4.98	67.72 ± 5.28	71.46 ± 2.74	72.36 ± 2.86	74.66 ± 4.04	75.54 ± 1.61	78.06 ± 1.69
Qubit = 7							
SVM	69.23 ± 5.28	76.42 ± 4.60	78.11 ± 3.79	78.57 ± 2.23	79.12 ± 2.40	80.39 ± 1.84	81.95 ± 1.19
QSVM	65.76 ± 5.17	69.17 ± 3.75	75.45 ± 3.09	76.62 ± 2.59	76.17 ± 2.96	79.13 ± 1.72	81.82 ± 1.47
Qubit = 9							
SVM	71.93 ± 4.04	78.59 ± 4.35	79.70 ± 3.21	79.92 ± 2.16	80.00 ± 2.18	81.40 ± 1.51	83.17 ± 1.38
QSVM	71.05 ± 6.10	69.32 ± 3.44	78.73 ± 2.94	77.21 ± 3.31	79.44 ± 2.87	79.53 ± 1.61	82.73 ± 1.13
Qubit = 11							
SVM	74.23 ± 4.87	78.16 ± 3.87	79.70 ± 3.21	80.37 ± 2.16	80.87 ± 2.24	81.52 ± 1.61	-
QSVM	72.69 ± 6.10	72.12 ± 3.10	78.73 ± 2.94	79.35 ± 1.86	77.98 ± 1.86	79.70 ± 1.90	-

SVM: Support Vector Machine, QSVM: Quantum Support Vector Machine.

**Table 3:** Comparisons of quantum support vector machine (QSVM) and support vector machine (SVM) classifier performance metrics, detailed.

Classifier		Performance Metrics (%)			
	Class	Precision	Sensitivity	Specificity	F1-Score
QSVM	AFIB	61.53	50.52	92.82	55.48
	GSVT	77.41	82.05	92.83	79.66
	SB	90.22	97.82	93.98	93.86
	SR	81.69	77.67	95.07	79.62
SVM	AFIB	57.25	37.36	91.92	45.21
	GSVT	71.85	82.90	87.58	76.98
	SB	89.62	98.64	91.21	93.91
	SR	6.38	5.55	94.44	5.93

QSVM: Quantum Support Vector Machine, SVM: Support Vector Machine, AFIB: Atrial Flutter (AF), Atrial Fibrillation (AFIB), GSVT: Supraventricular Tachycardia (SVT) + Atrial Tachycardia (AT) + Sinus Atrium to Atrial Wandering Rhythm (SAAWR) + Sinus Tachycardia (SINT) + Atrioventricular Node Reentrant Tachycardia (AVNRT) + Atrioventricular Reen-trant Tachycardia (AVRT), SB: Sinus Bradycardia, SR: Sinus Rhythm

**Table 4:** Performance metrics of QSVM with the highest accuracy and lowest accuracy.

Classifier		Performance Metrics (%)			
	Class	Precision	Sensitivity	Specificity	F1-Score
QSVM(H)	AFIB	75.34	53.65	95.56	62.67
	GSVT	77.73	90.05	93.15	83.43
	SB	95.51	100.00	96.89	97.70
	SR	79.39	81.44	95.01	80.40
QSVM(L)	AFIB	38.09	38.09	84.33	38.08
	GSVT	-	0	-	84.61
	SB	55.71	97.50	51.56	70.90
	SR	23.07	11.11	87.01	14.99

QSVM(H): Quantum Support Vector Machine (highest accuracy), QSVM(L): Quantum Support Vector Machine (lowest accuracy), AFIB: Atrial Flutter (AF), Atrial Fibrillation (AFIB), GSVT: Supraventricular Tachycardia (SVT) + Atrial Tachy-cardia (AT) + Sinus Atrium to Atrial Wandering Rhythm (SAAWR) + Sinus Tachycardia (SINT) + Atrioventricular Node Reentrant Tachycardia (AVNRT) + Atrioventricular Reentrant Tachycardia (AVRT), SB: Sinus Bradycardia, SR: Sinus Rhythm

**Table 5:** Comparison of some studies with Chapman dataset and some metrics of the proposed study.

Reference	Classifier	Accuracy (%)	F1—Score	Sensitivity	Specificity
Aziz et al. [29]	SVM MLP	84.2 90.7	- -	- -	- -
Sepahvand et al. [30]	Teacher Model CNN Student Model CNN	98.96 98.13	98.65 96.47	98.01 95.82	98.00 97.86
Faust et al. [31]	ResNet	99.98	-	99.94	100.00
Dhananjay et al. [32]	SVM CatBoost	71.00 99.00	66.11 99.00	72.50 99.17	- -
Murat et al. [18]	K-NN	80.94	77.92	78.03	93.75
	SVM	84.06	80.49	81.13	94.77
	RF	90.30	88.52	88.65	96.86
	NB	79.90	75.71	76.42	93.38
	GBC	87.68	85.21	85.53	96.03
	ABC	77.27	72.81	73.36	92.72
	DTC	85.78	83.46	83.54	95.41
	MLP	77.71	74.20	75.34	92.76
	QDA	77.01	72.79	73.62	92.44
Baygin et al. [33]	SVM	97.18	-	-	-
Proposed Method	SVM QSVM	86.96 84.64	82.41 81.15	81.70 81.13	95.61 95.00

SVM: Support Vector Machine, MLP: Multi-Layer Perceptron, CNN: Convolutional Neural Network, ResNet: Residual Network, K-NN: K-Nearest Neighbours, RF: Random Forest, NB: Naïve Bayes, GBC: Gradient Boosting Classifiers, ABC: AdaBoost Clas-sifier, DTC: Decision Tree Classifiers, QDA: Quadratic Discriminant Analysis, QSVM: Quantum Support Vector Machine.

## Data Availability

The ECG data in the study are the original data from the article by Zheng et al. (2020) [15]. All data can be accessed publicly at https://doi.org/10.6084/m9.figshare.c.4560497, accessed on: 8 February 2022.
